# Eosinopenia as Predictor of Disease Severity in Patients With Community-Acquired Pneumonia: An Observational Study

**DOI:** 10.1016/j.chest.2024.05.041

**Published:** 2024-07-05

**Authors:** Barbara Christine Weckler, Hendrik Pott, Alan Race, Nattika Jugkaeo, Kapil Karki, Stephan Ringshandl, Christian Seidemann, Ines Schöndorf, Harald Renz, Sebastian Fähndrich, Anna Lena Jung, Wilhelm Bertrams, Adeline Makoudjou, Daniela Zöller, Susetta Finotto, Stefanie Schild, Susanne A. Seuchter, Gernot Rohde, Frederik Trinkmann, Timm Greulich, Claus Franz Vogelmeier, Bernd Schmeck

**Affiliations:** a Department of Medicine, Pulmonary and Critical Care Medicine, Clinic for Airway Infections, University Medical Centre Marburg, Philipps-University Marburg, Marburg, Germany; b Institute for medical bioinformatics and biostatistics), Philipps-University Marburg, Marburg, Germany; c Department of Medicine, Data Integration Centre (DIC), Philipps-University Marburg, Marburg, Germany; d CALM-QE network; e SAPERCO GmbH; f Institute of Laboratory Medicine, Philipps University Marburg, Member of the German Centre for Lung Research (DZL), Member of Universities Giessen and Marburg Lung Centre, Marburg, Germany; g Department of Pneumology, Faculty of Medicine, University Medical Centre, Freiburg, Germany; h Institute for Lung Research, Universities of Giessen and Marburg Lung Centre, Marburg, Germany; i Core Facility Flow Cytometry—Bacterial Vesicles, Philipps-University Marburg, Marburg, Germany; j Institute of Medical Biometry and Statistics, Faculty of Medicine and Medical Centre, University of Freiburg, Freiburg, Germany; k Freiburg Centre for Data Analysis and Modelling, University of Freiburg, Freiburg, Germany; l Department of Molecular Pneumology, Friedrich-Alexander-University (FAU) Erlangen-Nürnberg University of Erlangen, Erlangen, Germany; m Medical Centre for Information and Communication Technology, University Hospital Erlangen, Erlangen, Germany; n Goethe University Frankfurt, University Hospital, Medical Clinic I, Department of Respiratory Medicine, Frankfurt/Main, Germany; o Department of Pneumology and Critical Care Medicine, Thoraxklinik, University of Heidelberg, Heidelberg, Germany; p Translational Lung Research Centre Heidelberg, German Centre for Lung Research, Heidelberg, Germany; q Department of Biomedical Informatics, Heinrich-Lanz-Centre, University Medical Centre Mannheim, Mannheim, Germany; r Department of Medicine, Pulmonary and Critical Care Medicine, University Medical Centre Marburg, Philipps-University Marburg, Marburg, Germany; s German Centre for Lung Research (DZL), Marburg, Germany; t German Centre for Lung Research (DZL) and Member of the German Centre of Infectious Disease Research, Marburg, Germany; u Centre for Synthetic Microbiology (Synmikro), Philipps-University Marburg, Marburg, Germany

To the Editor:

Lower respiratory tract infections, including community-acquired pneumonia (CAP), are a frequent cause of death worldwide.^1^ A previous study^2^ found that 38% of patients with CAP requiring mechanical ventilation did not survive their hospital stay. Conversely, only 16% of patients with CAP dying in hospital have been mechanically ventilated.^2^ Predictors of disease severity are required, because they would help identify high-risk patients with CAP who may benefit from early continuous monitoring to detect complications and reduce mortality. Previous studies found that eosinophil levels < 50/μL correlate with an increased mortality in hospitalized patients with CAP in addition to acute exacerbation of COPD.^3^ Also, eosinopenia is associated with a higher 4-week mortality in COVID-19 pneumonia.^4^ However, the role of eosinopenia for predicting short-term outcome in CAP is poorly understood.

This study therefore analyzed blood eosinopenia ≤ 50/μL as a predictor of disease severity in patients with CAP.

## Methods

We retrospectively reviewed ≥ 18-year-old patients hospitalized with CAP as their primary diagnosis from five university hospitals in Germany between 2009 and 2020. The diagnosis of CAP was based on the International Classification of Diseases Code J10-18 and its subgroups according to the 10th revision, German Modification.^5^

This study analyzed age, sex, and comorbidities including sepsis based on International Classification of Diseases, 10th Revision, German Modification^5^ in patients with eosinopenia ≤ 50/μL and non-eosinopenia (> 50/μL). Laboratory parameters were measured during the first 24 hours of admission. If multiple laboratory values were available for a single parameter, the first value was used for analysis. Mortality, need for both noninvasive and invasive mechanical ventilation in all patients, length of mechanical ventilation in survivors, date and time of hospital admission in all patients, and discharge in survivors or death of deceased patients were recorded based on the data in the hospital information system.

Data extraction and handling was performed under the umbrella of the Medical Informatics in Research and Care in University Medicine consortium.^6^ The study protocol was approved by all five local ethics committees, data privacy advocates, and the use and access committee. Data analysis was performed using DataSHIELD,^7^ an open-source software allowing privacy-preserving federated learning and anonymous co-analysis of individual-level data held at multiple locations. Descriptive statistics were reported as sample size-weighted average of the site-specific medians, and 25th and 75th percentiles. Percentages were used to report categorical variables distributions.

Differences in the comorbidity prevalence between the eosinopenia and non-eosinopenia groups were calculated via Fisher exact test in a univariate model. Differences in mortality and the need for mechanical ventilation between the two groups were analyzed using a multivariate general linear model that considered the following variables: age; sex; and blood values for C-reactive protein, creatinine, and hemoglobin in the eosinopenia and non-eosinopenia group.

Differences in length of stay in survivors, time to in-hospital death, and the number of hours of mechanical ventilation in survivors between the two groups were determined using Mann-Whitney *U* test^8^ in a univariate model. For reasons of data protection and technical requirements of the analysis software DataSHIELD, Mann-Whitney *U* test was modified as follows: Integer values were exported, decimal places were replaced by uniformly randomized numbers, and significance was subsequently calculated. This was done with N = 10,000, and the least significant *P* values were reported.

The statistical analysis was conducted using DataSHIELD version 6.1.1 and R versions 4.1.2 and 4.2.0. Nonadjusted *P* < .05 was considered statistically significant.

## Results

Overall, 6,748 (4,060 eosinopenic and 2,688 non-eosinopenic) patient cases were included in the analysis. Demographic data, laboratory parameters, and comorbidities of the eosinopenia and non-eosinopenia group are shown in Table 1.

**Table 1:** Baseline Characteristics in the Eosinopenia and Non-Eosinopenia Group

Variable	Eosinopenia Group (n = 4,060)	Non-Eosinopenia Group (n = 2,688)
Median age in years (25th-75th percentile)	71.08 (58.2-80.0)	69.45 (55.78-78.88)
Male sex, No. (%)	2,483 (61.2)	1,632 (60.7)
C-reactive protein, No. (%)	4,021 (99.0)	2,637 (98.1)
Median (mg/L), 25th-75th percentile (mg/L)	94.09, 39.61-175.92	78.92, 28.12-148.79
Procalcitonin, No. (%)	1,907 (47.0)	1,033 (38.4)
Median (ng/mL), 25th-75th percentile (ng/mL)	0.49, 0.23-1.76	0.27, 0.12-0.65
Leukocytes, No. (%)	4,060 (100)	2,688 (100)
Median (×10^9^/L), 25th-75th percentile (×10^9^/L)	9.76, 6.33-14.35	10.19, 7.46-13.61
Hemoglobin, No. (%)	4,059 (100)	2,688 (100)
Median (g/dL), 25th-75th percentile (g/dL)	12.43, 10.73-13.94	12.14, 10.5-13.7
Hematocrit, No. (%)	4,059 (100)	2,688 (100)
Median (%), 25th-75th percentile (%)	36.81, 32.31-40.87	36.51, 31.66-40.66
Creatinine, No. (%)	4,041 (99.5)	2,662 (99.0)
Median (mg/dL), 25th-75th percentile (mg/dL)	1.04, 0.8-1.49	1.02, 0.77-1.52
Asthma, No. (%)	87 (2.1)	76 (2.8)
COPD^a^, No. (%)	577 (14.2)	452 (16.8)
Congestive heart failure, No. (%)	801 (19.7)	503 (18.7)
Myocardial infarction^b^, No. (%)	66 (1.6)	66 (2.5)
Peripheral vascular disease^c^, No. (%)	292 (7.2)	242 (9.0)
Cerebrovascular disease, No. (%)	242 (6.0)	154 (5.7)
Dementia, No. (%)	340 (8.4)	190 (7.1)
Diabetes mellitus, No. (%)	966 (23.8)	634 (23.6)
Liver disease, No. (%)	146 (3.6)	95 (3.5)
Renal disease^d^, No. (%)	880 (21.7)	697 (25.9)

Significant differences between eosininopenia and non-eosinopenia group:

a *P* = .004;

b *P* = .019;

c *P* = .008;

d *P* < .0001.

**Figure 1:**
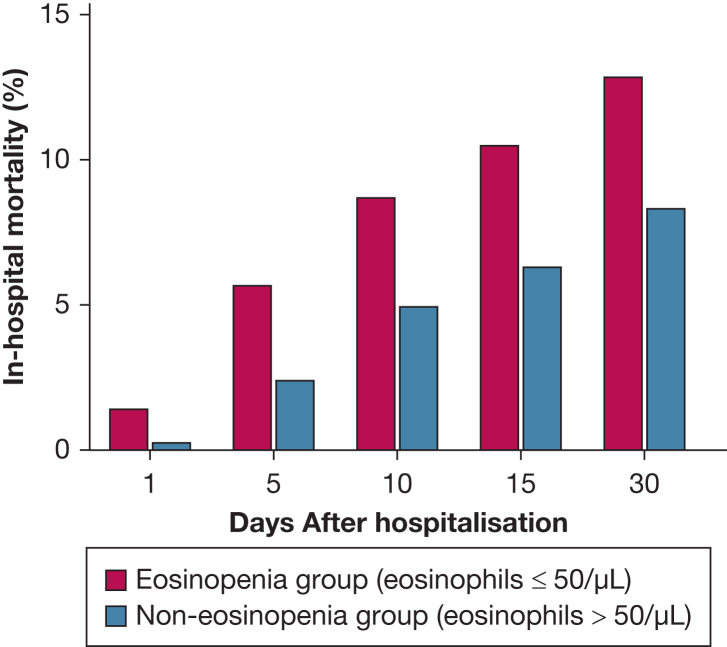
In-hospital mortality in the eosinopenia vs non-eosinopenia group on days 1, 5, 10, 15, and 30 after hospitalization.

In-hospital mortality was significantly higher in the eosinopenia vs non-eosinopenia group (13.8% vs 9.1%; relative risk [RR], 1.51; 95% CI, 1.31-1.74; *P* < .0001). In-hospital mortality in the two groups on days 1, 5, 10, 15, and 30 after hospitalization is displayed in Figure 1. The need for mechanical ventilation was significantly elevated in the eosinopenia vs non-eosinopenia group (19.2% vs 14.3%; RR, 1.34; 95% CI, 1.20-1.50; *P* < .0001). Risk of sepsis was significantly elevated in the eosinopenia vs non-eosinopenia group (7.5% vs 5.0%; RR, 1.50; 95% CI, 1.23-1.83; *P* < .0001). Median length of stay in eosinopenic survivors was significantly prolonged compared with non-eosinopenic survivors (8.41 vs 7.64 days; *P* < .0001). Median time to in-hospital death was significantly reduced in the eosinopenia group (6.73 vs 8.92 days; *P* = .001). In survivors, median length of mechanical ventilation in the eosinopenia group was 121.93 hours vs 93.39 hours in the non-eosinopenia group. The difference was not significant (*P* = .152).

## Discussion

The key finding of this study was an association between blood eosinopenia (≤ 50/μL) and an increase in in-hospital mortality, need for mechanical ventilation, risk of sepsis, length of stay in survivors, and reduced time to in-hospital death in a real-world patient cohort.

Because the prevalence of COPD, myocardial infarction, peripheral vascular disease, and renal disease was lower in the eosinopenia group, whereas the other comorbidities were balanced in the eosinopenia and non-eosinopenia groups, the worse outcomes in eosinopenic patients do not seem to be caused by comorbidities.

In line with our findings, eosinopenia < 50/μL correlates with an increased 18-month mortality in hospitalized patients with acute exacerbations of COPD and CAP,^3^ and, as part of the Dyspnoea, Eosinopenia, Consolidation, Acidaemia, and Atrial Fibrillation score,^9^ elevated short-term mortality in patients with acute exacerbations of COPD. Furthermore, eosinopenia is associated with a higher 4-week mortality in COVID-19 pneumonia.^4^

Solid data about eosinophil numbers as a predictor of short-term outcome in CAP in humans are missing. In a mouse model,^10^ after induction of pneumonia with *Staphylococcus aureus*, an IL-33-induced increase in eosinophil levels inhibited acute lung injury as indicated by reduced pulmonary edema and higher oxygen saturations. This was associated with improved survival. Eosinophil reduction prevented IL-33-mediated protection against mortality in mice. Future studies considering eosinophil and IL-33 levels, as well as downstream signaling pathways, are needed to elucidate the mechanism by which eosinophils might benefit survival after CAP.

The strengths of our study relate to the high number of real-world patient cases.

The limitations of this study involve its retrospective design; that its analysis was based on cases instead of patients; the lack of information about the immune status, microbial etiology, and clinical parameters of patients; and the technical restrictions of DataSHIELD, which did not allow for propensity score matching and analyzing eosinophil count as continuous variables.

In conclusion, blood eosinopenia ≤ 50/μL (vs non-eosinopenia) seems suitable to predict disease severity in patients with CAP. Further prospective studies are required to confirm our findings.

## Funding/Support

This work was funded in part by the Bundesministerium für Bildung und Forschung (Federal Ministry of Education and Research; BMBF), MIRACUM within the Medical Informatics Funding Scheme (FKZ 01ZZ1606A-H) to H.R. and C.V.; PermedCOPD—FKZ 01EK2203A to C. V. and B. S.; CALM-QE—FKZ 01ZZ2318A to B. S. and H. R.; the Deutsche Forschungsgemeinschaft (SFB/TR-84 TP C01), and the von-Behring-Röntgen-Stiftung (66-LV07) to B. S., and the Hessisches Ministerium für Wissenschaft und Kunst (LOEWE Diffusible Signals) to A. L. J. and B. S.

## Financial/Nonfinancial Disclosures

The authors have reported to *CHEST* the following: B. C. W. reports one unpaid participation in an Advisory Board with AstraZeneca. A. L. J. reports research grants from the Behring Röntgen Foundation. G. R. reports personal fees from Astra Zeneca, Atriva, Boehringer Ingelheim, GSK, Insmed, MSD, Sanofi, Novartis, and Pfizer for consultancy during advisory board meetings and personal fees from Astra Zeneca, Berlin Chemie, BMS, Boehringer Ingelheim, Chiesi, Essex Pharma, Grifols, GSK, Insmed, MSD, Roche, Sanofi, Solvay, Takeda, Novartis, Pfizer, and Vertex for lectures including service on speakers’ bureaus. F. T. reports consulting fees from AstraZeneca, Berlin Chemie, Boehringer Ingelheim, Bristol-Myers Squibb, Chiesi, Fisher & Paykel, GlaxoSmithKline, Janssen-Cilag, Novartis, Omron, OM-Pharma, Roche, Sanofi-Aventis, and support for attending meetings or travel from AstraZeneca, Actelion, Bayer, Berlin Chemie, Boehringer Ingelheim, Chiesi, Mundipharma, Novartis, Pfizer, TEVA. T. G. reports Grant from Grifols to institution for AATD laboratory, consulting fees from AstraZeneca, Berlin-Chemie, Boehringer-Ingelheim, Chiesi, CSL-Behring, Grifols, GSK, Mundipharma, Novartis, Takeda, payment or honoraria for lectures, presentations, speakers bureaus, manuscript writing or educational events from AstraZeneca, Berlin-Chemie, Boehringer-Ingelheim, Chiesi, CSL-Behring, Grifols, GSK, Mundipharma, Takeda, support for attending meetings or travel from AstraZeneca, Berlin-Chemie, Chiesi, CSL-Behring, Grifols, GSK, Novartis, participation on a Data Safety Monitoring Board or Advisory Board with AstraZeneca, Berlin-Chemie, Boehringer-Ingelheim, Chiesi, CSL-Behring, Grifols, GSK, Mundipharma, Novartis, Takeda, leadership or fiduciary role in other board, society, committee or advocacy group, paid or unpaid as scientific Advisor for Alpha-1-Deutschland. C. F. V. reports grants or contracts from German Ministery of Education and Science (BMBF), AstraZeneca, Boehringer Ingelheim, Chiesi, CSL Behring, GlaxoSmithKline, Grifols, Novartis, consulting fees from Aerogen, AstraZeneca, Boehringer Ingelheim, CSL Behring, Chiesi, GlaxoSmithKline, Insmed, Menarini, Novartis, Nuvaira, Sanofi, payment or honoraria for lectures, presentations, speakers bureaus, manuscript writing or educational events from Aerogen, AstraZeneca, Boehringer Ingelheim, CSL Behring, Chiesi, GlaxoSmithKline, Insmed, Menarini, Novartis, Roche, Sanofi. Bernd Schmeck reports Grants or contracts from CSL-Behring. None declared (H. P., A. R., N. J., K. K., S. R., C. S., I. S., H. R., S. Fähndrich., W. B., A. M., D. Z., S. Finotto., S. S., S. A. S., B. S.).
